# A. Kocher technique as default approach to aortic root enlargement

**DOI:** 10.1016/j.xjtc.2026.102296

**Published:** 2026-03-02

**Authors:** Alfred A. Kocher, Paul Werner, Emilio Osorio-Jaramillo, Amila Kahrovic, Daniel Zimpfer, Marek Ehrlich, Severin Laengle

**Affiliations:** Department of Cardiac and Thoracic Aortic Surgery, Medical University of Vienna, Vienna, Austria

**Figure:**
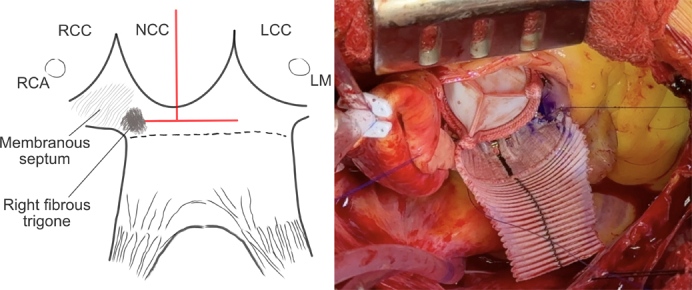
Inverted, asymmetric T-incision (L), barrel-shaped aortic patch for root enlargement (R).

**Central Message**

The novel, eclectic A. Kocher technique presents a simplified, broadly applicable root enlargement procedure.

Implantation of a small aortic valve prosthesis should be avoided because it carries the risk of patient-prosthesis mismatch (PPM), a known risk factor for worse clinical outcomes. Considering the importance of lifetime management in aortic valve disease, larger prostheses provide a superior platform for potential future valve-in-valve procedures. The study was approved by the institutional review board (EK 1180/2019, approved April 16, 2019). Informed consent was waived.

Several techniques have been described for surgical enlargement of the aortic annulus and root. Soon after the first orthotopic aortic valve replacement by Harken in 1960, Nicks and colleagues^1^ proposed an enlargement via extension of the aortotomy through the noncoronary sinus across the aortic annulus into the base of the anterior mitral valve leaflet and subsequent patch augmentation, which allows an increase by one valve size. Manouguian and Seybold-Epting^2^ proposed a posterior incision from the top of the left-non commissure towards the mitral valve and halfway through its anterior leaflet. Nunez modified the technique by limiting the incision to the subaortic curtain. Conversely, the Rastan-Konno procedure is a true annular and left ventricular outflow tract enlargement, often applied in congenital aortic valve stenosis.^3^

Recently the topic has gained traction with the Y-incision proposed by Bo Yang.^4^ This approach allows for up to 4 sizes bigger prostheses in the majority of patients. In contrast to other procedures, the incision is Y-shaped, spanning one third of the annular circumference, thereby creating a larger defect that is addressed by a rectangular patch, centered in the left-non commissure.

Prompted by the very important work by Yang and colleagues, we created a novel surgical procedure - the A. Kocher technique—to simplify aortic root enlargement and facilitate a broader applicability.^5^ Herein, we present the technique and the initial series of patients.

## Surgical Technique

At our institution, the avoidance of PPM is a top priority. To succeed with this goal, we created a novel technique that provides a reliable root enlargement to accommodate valve prostheses that are at least 2 sizes bigger. This technique requires neither previous planning nor a special surgical approach to the aortic valve.

**Figure 1:**
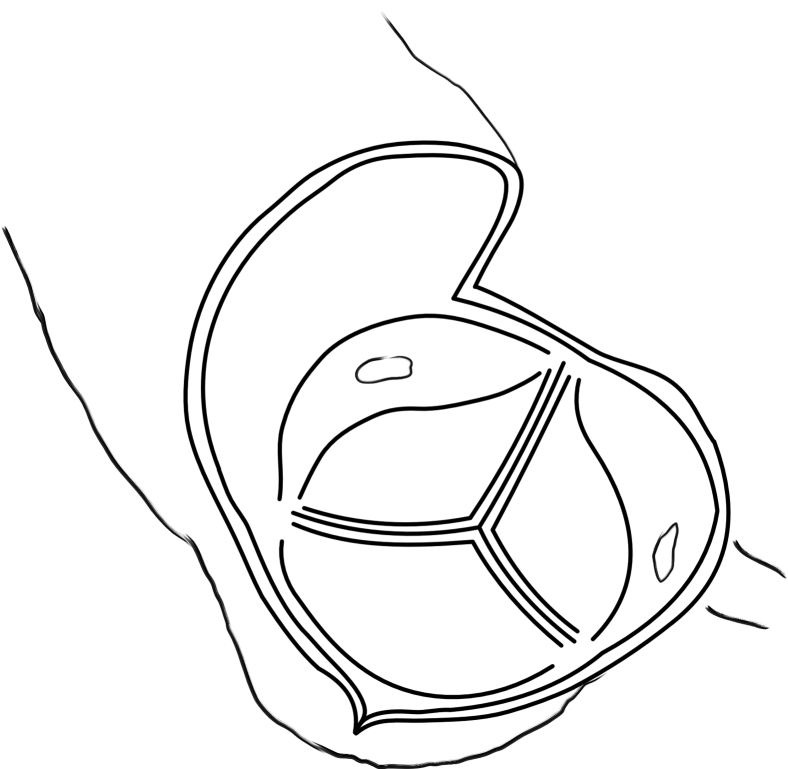
Initial hockey-stick aortotomy reaching into the noncoronary sinus.

**Figure 2:**
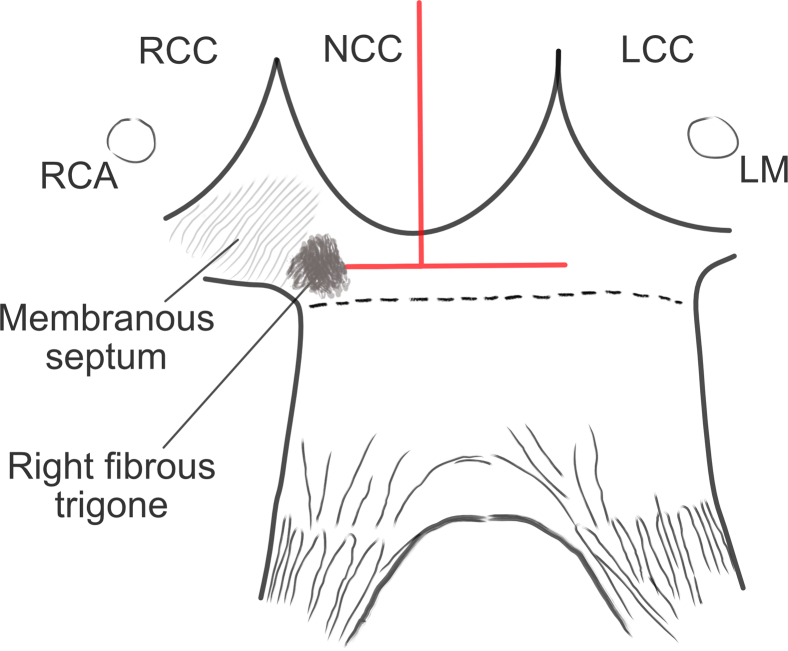
Asymmetric inverted T incision for root enlargement. *RCA*, Right coronary artery; *RCC*, right coronary cusp; *NCC*, noncoronary cusp; *LCC*, left coronary cusp; *LM*, left main.

**Figure 3:**
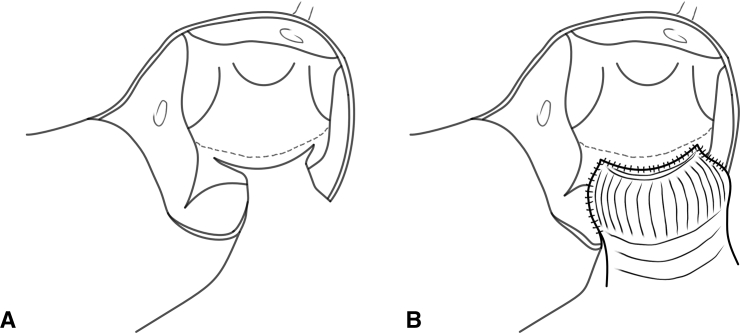
A, Top view on the aortic root after performing the asymmetric inverted T incision. B, View on the root after implantation of the barrel-shaped patch.

**Figure 4:**
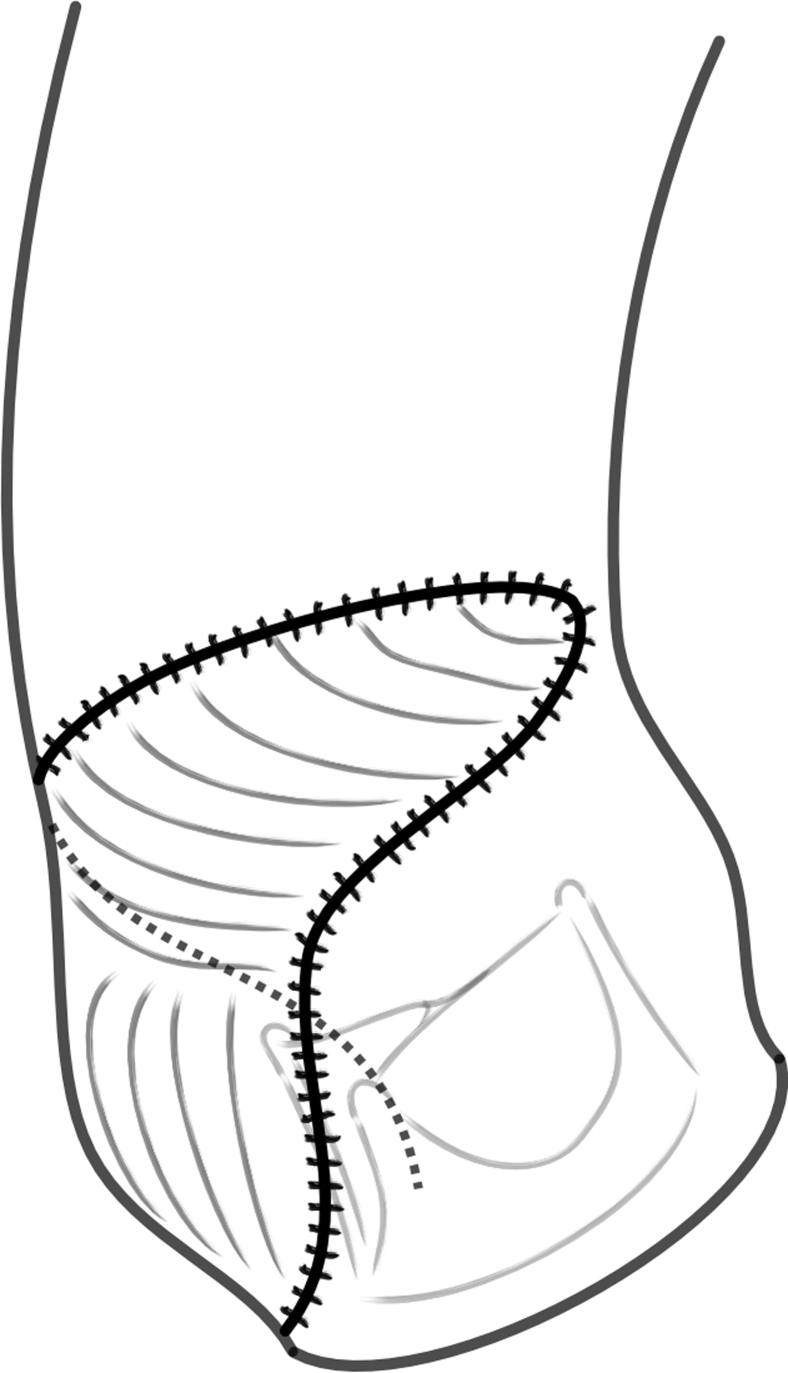
The closed ascending aorta with the integrated patch.

A typical hockey-stick incision that extends to the top of the center of the noncoronary sinus is made (Figure 1). In case enlargement is required, the A. Kocher technique is carried out after sizing: The incision is continued toward the nadir of the noncoronary sinus. At the level of the basal ring, the inverted asymmetric T-incision is extended alongside the hinge line of the anterior mitral valve leaflet to the center of the base of the left noncommissure and in the opposite direction limited to a few millimeters toward the right fibrous trigone (Figure 2). Care is taken not to open the membranous septum. The length of the transverse section at the annular level—usually 2.5 to 3 cm—is measured, and a corresponding polytetrafluoroethylene patch is tailored form a 34-mm Valsalva prosthesis to preserve the curvature of the aortic annulus and create an anatomically shaped noncoronary neosinus (Figure 3). The patch is barrel-shaped, with the widest part at the midaortic sinus level and sewn in with a 4-0 polypropylene suture in a running technique. The valve implantation is performed in a standard fashion and the aortotomy is closed including the patch (Figure 4).

**Figure 5:**
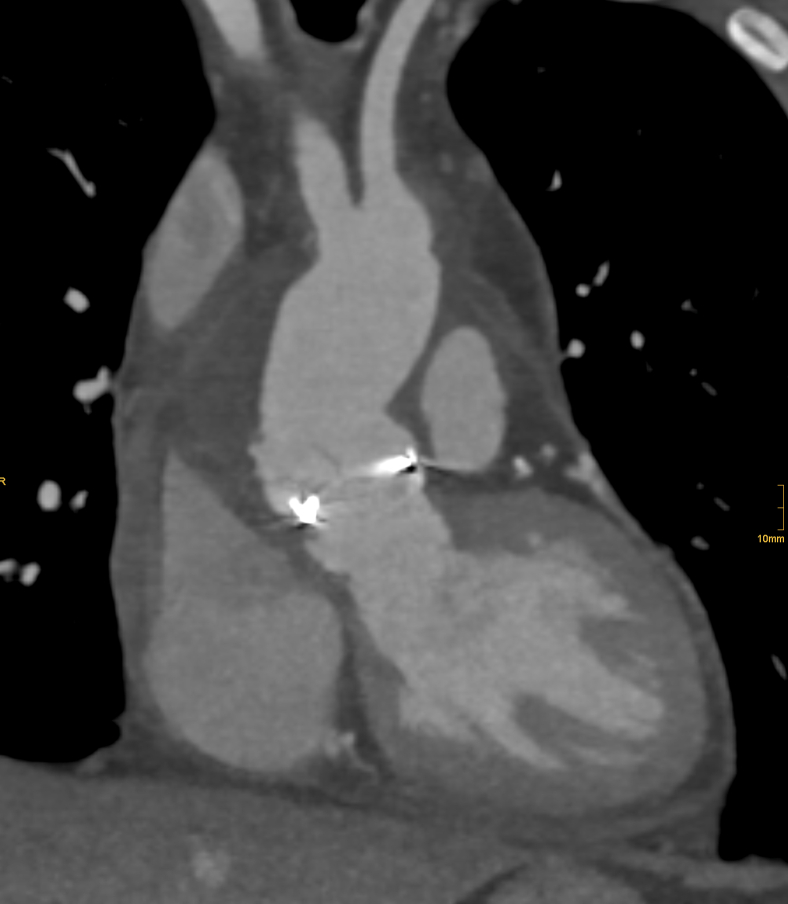
Frontal plane of postoperative CT scan after aortic valve replacement and A. Kocher root enlargement.

### Case Series

We performed the A. Kocher procedure in 13 patients. Twelve patients presented with significant aortic valve stenosis and 1 patient with severe regurgitation. Operative characteristics and outcome are summarized in Table 1. Median duration of surgery was 275 (61) minutes, with a median crossclamp time of 113 (35) minutes. Initial intraoperative measurement revealed annular diameters between 17 and 21 mm. Eleven patients received a tissue and two a mechanical prosthesis, with sizes ranging from 21 to 29 mm. Technical success of annular enlargement was achieved in the entire cohort, with a survival of 100% to the latest follow-up.

**Table 1:** Patient and operative characteristics

Case	Sex	Diagnosis	BSA, m^2^	Annulus size	Prosthesis	Prosthesis size	Total time, min	CPB time, min	CX time, min	Mitral regurgitation (0/1/2/3)	AV-block III (Y/N)	Death (Y/N)
1	Female	AS	1.62	21	Tissue	25	300	176	113	0	N	N
2	Female	AS	1.61	19	Mechanical	23	268	182	139	2	N	N
3	Male	AS	1.96	21	Mechanical	25	251	123	84	0	N	N
4	Female	AR	1.58	17	Tissue	21	365	207	130	0	N	N
5	Female	AS	1.74	21	Tissue	25	261	123	95	0	N	N
6	Female	AS	1.6	19	Tissue	23	233	140	118	0	N	N
7	Male	AS	1.97	21	Tissue	25	222	137	106	1	N	N
8	Female	AS	1.94	17	Tissue	21	305	172	137	1	N	N
9	Female	AS	1.49	19	Tissue	23	273	125	103	2	N	N
10	Female	AS	1.88	19	Tissue	23	351	198	130	1	N	N
11	Male	AS	2.17	21	Tissue	25	337	167	133	0	N	N
12	Male	AS	2.28	23	Tissue	29	199	92	77	0	N	N
13	Female	AS	1.75	19	Tissue	25	297	229	116	2	N	N
14	Male	AS	1.95	23	Tissue	27	293	98	79	0	N	N

*BSA*, Body surface area; *CPB*, cardiopulmonary bypass; *CX*, crossclamp; *AV*, atrioventricular; *Y*, yes; *N*, no; *AS*, aortic valve stenosis; *AR*, aortic valve regurgitation.

## Discussion

The term PPM in valve surgery was coined in 1978, even though the concept was well known and existed as a clinical problem from the get-go of surgical aortic valve replacement. As PPM results in poorer clinical outcomes, a number of both root and annular enlargement techniques have been developed over the past decades. These techniques have never gained widespread application because of the surgical complexity and the associated risk of the procedure. Although robust evidence of the benefit of root enlargement compared with isolated valve replacement is still lacking, the role of root enlargement for lifetime management and avoidance of PPM has been recognized by international valve guidelines.

The recently described procedure by Bo Yang renewed interest in this topic. The enlargement technique advanced and refined at our institution by A. Kocher presents several distinct advantages:

1. Standard aortotomy: A routine hockey-stick incision can be performed. The decision to enlarge the aortic root can be made after sizing of the annulus.
2. Coronary arteries are not compromised: The inverse asymmetric T-incision along the hinge line of the mitral valve is limited to the center of the base of the left noncommissure, which leaves the area of the left coronary cusp completely untouched, thus preventing any distortion of the left main stem. The preserved distance to both coronary ostia is particularly important in view of the lifetime management of patients with implanted aortic valves.
3. Taking the patch from a Valsalva prosthesis not only preserves the circular anatomy of the aortic annulus but also provides an anatomical noncoronary neosinus and increases the cubic volume of the aortic root.
4. As opposed to previously described aortic root enlargement approaches with the A. Kocher technique, the suture lines are confined to the noncoronary sinus and therefore easier accessible.
5. The patch enlargement in the noncoronary sinus results in preservation of the natural geometry of the left and right sinuses and commissures and allows for a near-anatomical positioning of the valve prosthesis resulting in an axial flow pattern (Figure 5).

In summary, the proposed A. Kocher technique is a novel root-enlargement procedure incorporating several aspects of the previously described techniques in an eclectic fashion. Our initial experience revealed an easy reproducibility with an implantation of at least 2 valve sizes bigger prosthesis reducing the risk of PPM.

## Conflict of Interest Statement

The authors reported no conflicts of interest.

The *Journal* policy requires editors and reviewers to disclose conflicts of interest and to decline handling or reviewing manuscripts for which they may have a conflict of interest. The editors and reviewers of this article have no conflicts of interest.
